# Implementation and Analysis of Real-Time Streaming Protocols

**DOI:** 10.3390/s17040846

**Published:** 2017-04-12

**Authors:** Iván Santos-González, Alexandra Rivero-García, Jezabel Molina-Gil, Pino Caballero-Gil

**Affiliations:** Department of Computer Engineering and Systems, University of La Laguna, 38206 Tenerife, Spain

**Keywords:** streaming, quality of experience, quality of service, Android, WebRTC, RTSP

## Abstract

Communication media have become the primary way of interaction thanks to the discovery and innovation of many new technologies. One of the most widely used communication systems today is video streaming, which is constantly evolving. Such communications are a good alternative to face-to-face meetings, and are therefore very useful for coping with many problems caused by distance. However, they suffer from different issues such as bandwidth limitation, network congestion, energy efficiency, cost, reliability and connectivity. Hence, the quality of service and the quality of experience are considered the two most important issues for this type of communication. This work presents a complete comparative study of two of the most used protocols of video streaming, Real Time Streaming Protocol (RTSP) and the Web Real-Time Communication (WebRTC). In addition, this paper proposes two new mobile applications that implement those protocols in Android whose objective is to know how they are influenced by the aspects that most affect the streaming quality of service, which are the connection establishment time and the stream reception time. The new video streaming applications are also compared with the most popular video streaming applications for Android, and the experimental results of the analysis show that the developed WebRTC implementation improves the performance of the most popular video streaming applications with respect to the stream packet delay.

## 1. Introduction

In recent years, the use of video content on the Internet has increased to 60% of all global traffic. Different studies, such as [1], predict that the Internet video traffic will account for 80 percent of all Internet consumer traffic in 2019. This rapid increase is partly due to the huge growth in the sales of smartphones over the last few years, as smartphones have become the major video content creators. For this reason, some of the most important multimedia content companies have tried to develop new strategies to improve the Quality of Experience (QoE) and the Quality of Service (QoS) of their systems. The rapid evolution of technology and the low cost of mobile devices make them excellent candidates for offering new streaming functionalities with different purposes. The life of people can change with simple streaming applications as some of the uses of this technology can turn people into potential journalists who use their smartphones to broadcast videos over the Internet, do telemedicine [2,3] and operate or assist somebody with the remote assistance of a specialist doctor, broadcast sports in real time or use this technology in e-learning [4].

In this work, the most used video streaming protocols and applications are studied. In particular, two video streaming platforms have been developed that implement different video streaming protocols in order to compare both protocols and conclude which of them offers the best results in the scope of Android and web real-time video streaming applications. The analysis of the implemented systems includes different parameters related to the QoS and QoE, namely the connection establishment time and the stream reception time. Although the QoE depends on the users in a subjective way, it depends directly on the QoS because a good QoS helps to obtain a better QoE by part of the user. For this reason, the parameters analysed in this work measure the QoS in a direct way, but indirectly influence the QoE. In addition, these two implementations were compared with the most widely used Android and web video streaming applications, which implement those and other protocols. In order to carry out such a comparison, a metric based on the stream delay time was used to know the efficiency of the implemented systems in relation to the most used systems today.

The present work is structured as follows. Section 2 describes a brief state-of-the-art. Section 3 introduces two of the most commonly used video streaming protocols, RTSP and WebRTC. The developed implementations are defined in Section 4. Section 5 presents the comparative studies between the implemented systems and the most used web and Android applications. Finally, some conclusions and open issues close the work in Section 6.

## 2. Related Work

In recent years, different proposals have been developed to measure and improve the QoS and QoE of video streaming systems.

One of the first works related to the topic is [5], which compares HyperText Transfer Protocol (HTTP), Real Time Streaming Protocol (RTSP) and InterMedia Streaming (IMS), and describes several approaches that promise the synthesis of networks based on those protocols. The main advantage of this proposal is the research of a future possible expansion of IMS to harmonize Session Initiation Protocol (SIP), HTTP, and RTSP service delivery, a fact that is related to the video streaming quality. However, that work studies only the cases of HTTP, RTSP and IMS, forgetting other promising protocols like WebRTC.

The subsequent work [6] also contains a study of HTTP, RTSP and Dynamic Adaptive Streaming over HTTP (DASH) [7] applied over smartphones. The method to analyse these protocols consists of the calculation of the switch delay or the time between the moments when a user sends a command and when the client screen undergoes these changes. The main contribution of that work is the use of metrics to compare the different video streaming protocols in the fields of bandwidth consumption and QoS. Its disadvantages compared with the present work are that, despite the relative youth of the proposal, the WebRTC protocol is not included, and the delays in the video streaming connection establishments are not compared.

A video streaming framework for QoE management of Scalable Video Coding (SVC) based on the H.264/SVC codec is presented in [8], together with a measurement study to quantify the influence of different parameters such as video resolution, scaling method, network conditions and video content type on the QoE of streaming. The main difference between that study and the one presented in this paper is that they considered that the most important measurements are those related to the video itself, while this work focuses on the protocols that send the video to other users.

In [9], the authors integrate the SVC extensions of the Advanced Video Coding (AVC) standard into the recently ratified MPEG-DASH standard to improve its performance. Moreover, they present a comprehensive study that evaluates the presented solution applying restricted conditions, and compare it with another version of MPEG-DASH implementation using AVC, which is the typical case in commercial applications. The main contribution of that work is that it presents a comparative study of the most used MPEG-DASH implementations using AVC. However, that work is focused only on one side of video streaming applications, which is the codec used in the video codification, while, in this paper, the main purpose is the study and comparison of the other side of video streaming applications, the communication protocol.

Subsequently, the work [10] describes different measurements collected from DASH and Web Real-Time Communication (WebRTC) implementations while moving at walking speeds through an 802.16e WiMAX network. The collected data come from an application, the network and the physical layers under different wireless environments. In addition, the work also identifies the features that directly affect the quality of the video service in the mobile data network, in order to conclude that in order to adapt the channel conditions, these services do not achieve acceptable quality of service for mobile users under different network conditions.

On the subject of the use of dynamic adaptive streaming over HTTP, which is a widespread topic in the field of video streaming systems, the work [11] presents a range of issues related to the performance of DASH, studying the most important parameters that affect this type of video streaming systems. The main contribution of this work is concretely the study of the DASH techniques and the parameters that affect the streaming QoE to understand in a better way how the DASH streaming techniques works. Despite the fact that that work only deals with DASH streaming techniques, the knowledge obtained from it will be important to compare the protocols used in different commercial applications and to extend the present paper to DASH streaming applications in the future.

In the recent proposal [12], a QoE instrumentation for video streaming on smartphones, called VLQoE, is presented. The authors use a VLC media player to record a set of metrics from the user interface, application-level, network-level and available sensors of the device, and present two state models based on the time of HTTP and RTSP protocols streaming via 3.5 G. The main contribution of that proposal is the creation of some objective metrics to measure the QoE independently of the user and its subjectivity in smartphones using both RTSP and HTTP streaming protocols through the use of the VLQoE tool-kit, which seems to be the first objective tool to measure the QoE. These metrics are a good starting point to understand better how important the QoE is for the final user.

Finally, a recent study on the QoE of three of the most common video streaming protocols is presented in [13]. The main purpose of that work is to measure the QoE of MPEG-DASH, Real Time Messaging Protocol (RTMP) and RTSP protocols by applying some non-intrusive methods to monitor video streaming under different network conditions. In practice, the RTMP and RTSP protocols are very similar, so the obtained results are also very similar. In addition, the MPEG-DASH protocol in the scope of media streaming, like could be the Video-on-demand (VoD) [14], is widely used. However, in the scope of video streaming communications, real-time communications over video are not used as much. As expected, RTSP offers slightly worse results compared to RTMP, but this is normal because the protocols are very similar and the RTSP protocol implements some mechanisms that make it a bit less efficient than the RTMP protocol. The main difference between that work and the one presented here is that such a work focuses on the QoE in VoD protocols, whereas, in the present work, the main purpose is the study of real-time streaming protocols.

## 3. Preliminaries

Choosing a streaming protocol is a difficult task that depends on the type of information to be shared. Communication must be made using a protocol formed by a group of rules defining how data are transmitted over the network and divided into different parts, such as headers, data, authentication and error handling. Thus, a streaming protocol may be viewed as a communication protocol where the transmitted data are media data.

In this study, the main objective is to share audio and video media. For this reason, the most important point is the guarantee of a low latency and efficient transmissions with occasional packet losses. A media streaming protocol is defined, taking into account the structure of the packets and the algorithms used to send real-time media on a network. Different media streaming protocols are available today, which differ in a few implementation details.

This section is focused on the study of two of the most used streaming protocols: RTSP and WebRTC, which are used as base of the platforms developed in this work. Traditionally, these communication categories have been divided into push-based and pull-based protocols [15].

Push-based protocols consist of established communication between the client and the server, where the client is responsible for establishing the connection, and the server sends a packet stream until the client stops or interrupts the communication. In this type of protocol, the server, in addition to sending media packets, maintains a session to listen for commands from the client. These protocols usually work over the User Datagram Protocol (UDP) [16], but could work over the Transmission Control Protocol (TCP) [17] if necessary. They normally use the Real-time Transport Protocol (RTP), specified in RFC 3550 [18].

Pull-based protocols are based on the HTTP protocol and consist of a communication between client and server where the client is responsible for sending a request to the server, and the server starts a communication where the client downloads the video streaming. In these protocols, the streaming speed depends on the bandwidth of the client network. The most commonly used pull-based protocol is a progressive download, in which a client sends a request to the server and starts pulling the media content from it as quickly as possible.

Table 1 shows some examples of streaming protocols and their features. This information is based on [19], where there is an analysis of the transmission protocols for the H.265 encoder.

The two most used real-time streaming protocols are explained in the following subsections.

### 3.1. RTSP

The RTSP [20] is a non-connection-oriented application layer protocol that uses a session associated with an identifier. RTSP typically applies the UDP protocol to share video and audio data, and TCP for control, if necessary. The syntax of the RTSP protocol is similar to that of the HTTP protocol and supports the following operations:

- Retrieval of media from media server: The client can request a presentation description via HTTP or some other method. If the presentation is multicast, the presentation description contains the multicast addresses and ports that will be used for the continuous media. If the presentation is to be sent only to the client via unicast, the client provides the destination for security reasons.
- Invitation of a media server to a conference: A media server can be “invited” to join an existing conference, either to play back media in the presentation or to record all or a subset of the media in a presentation. This mode is useful for distributing teaching applications. Several participants in the conference may take turns “pushing the remote control buttons”.
- Addition of media to an existing presentation: Particularly for live presentations, it is useful for the server to be able to inform the client about the availability of additional media.

The structure of a URL for RTSP is very similar to the URL in HTTP, with the only difference in the used scheme *rtsp://* in RTSP instead of *http://* in the HTTP protocol, and the addition of new request methods such as DESCRIBE, SETUP, PLAY, PAUSE and TEARDOWN. The DESCRIBE method is used to obtain a description of the presentation or object appointed by the URL RTSP in conjunction with the use of the Session Description Protocol. The server responds to this request with a description of the requested resource. This answer corresponds to the initialization phase of RTSP and contains the list of required multimedia streams. On the other hand, the SETUP method is used to establish how the stream is transported, the request contains the URL of the multimedia stream and a transport specification that usually includes the port to receive RTP video and audio data and another for the RTCP [21] meta-data. The server responds by confirming the selected parameters and fills the other parts, as they are the ports selected by the server. Every stream has to be configured before sending a PLAY request.

The PLAY request is used to start the data stream shipment by the server using the ports configured with the SETUP request method. Moreover, the PAUSE method temporarily pauses one or all streams to resume later with a PLAY request. Finally, the use of the TEARDOWN request method stops the shipment of data, releasing all resources. Note that, first of all, a TCP connection is established between the client and the server, started by the client and typically over a well-known TCP port (554).

**Figure 1:**
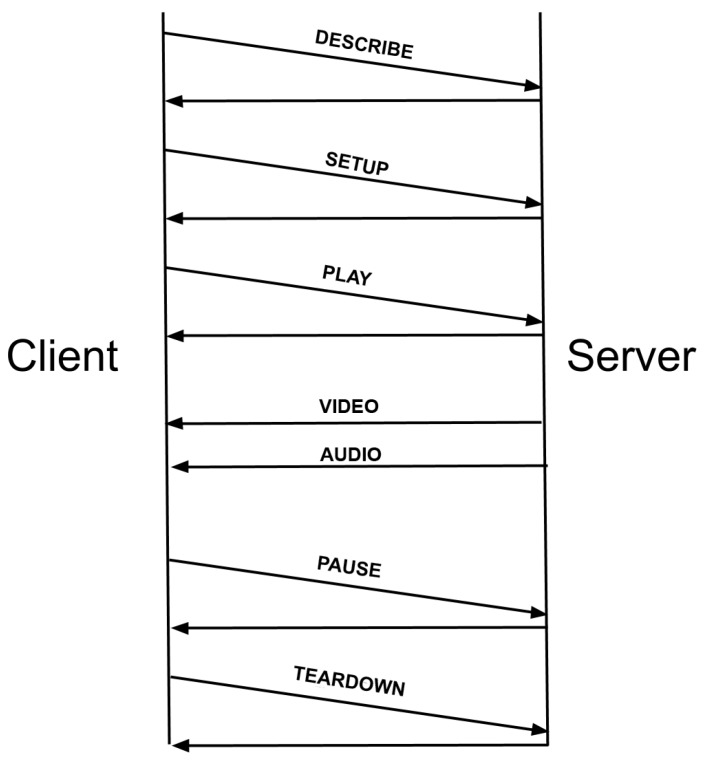
Real Time Streaming Protocol (RTSP) request order.

The operation and request order of the RSTP protocol are shown in Figure 1.

### 3.2. WebRTC

WebRTC [22] is an Application Programming Interface (API) created by the World Wide Web Consortium (W3C) that allows browser applications to make calls and video chats and to use P2P files without any plugin. The first implementation of WebRTC was created by Google and released as Open Source. Different bodies such as the Internet Engineering Task Force, created to standardize the used protocols, and the W3C, with the browser APIs, have been working on this implementation.

The main components of WebRTC are the following:

- getUserMedia: It allows obtaining video or audio streams from the microphone or camera hardware. This API can be used to get a screenshot or to share our screen with other users too.
- RTCPeerConnection: It allows setting up the audio/video stream. It consists of a lot of different tasks such as signal processing, codec execution, bandwidth administration, security of the streaming, etc. This API call can be used to implement these different tasks without the intervention of the programmer.
- RTCDataChannel: It allows sharing video or audio data between connected users. RTCDataChannel uses a two-way communication between peers, and can be used to exchange any data type. To do this, RTCDataChannel uses Websockets, which allows bidirectional communication between the client and the server, using either a slower and reliable communication over TCP, or a faster and non-reliable communication over UDP.
- geoStats: API call that allows getting different statistics about a WebRTC session.

**Figure 2:**
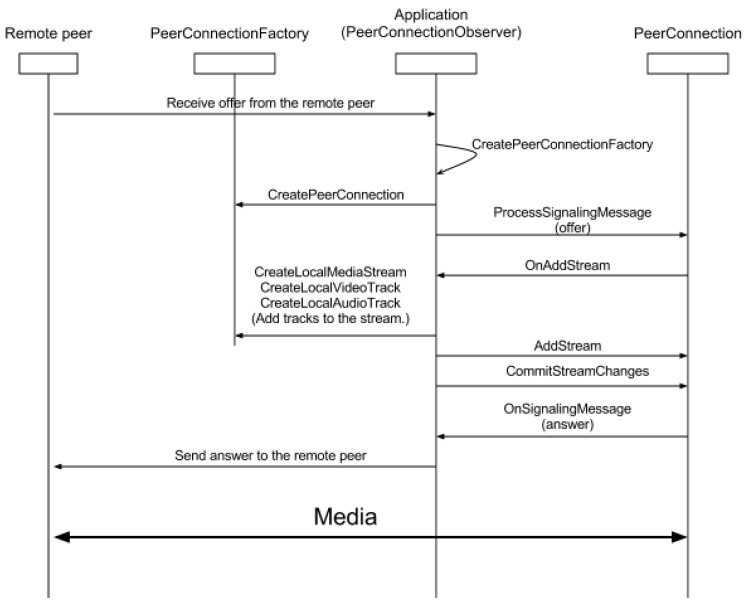
Web Real-Time Communication (WebRTC) request order.

Figure 2 shows the operation of the WebRTC protocol used to make a call with the aforementioned API, which is composed of the next four steps:

- The application receives a streaming offer from the RemotePeer.
- The next step is the instantiation of the PeerConnection by from the application.
- Once the PeerConnection is created, the application generates the media stream and the audio and video tracks through the PeerConnectionFactory and adds the created stream to the PeerConnection.
- Finally, the application responds to the RemotePeer and starts the media communication.

The security of the WebRTC protocol is based on two parts. On the one hand, it uses the Datagram Transport Layer Security (DTLS) protocol [23], which is based on the TLS protocol and involves a similar security level, in the previous communications, where the parameters of the media communications are established. On the other hand, the Secure Real-time Transport Protocol (SRTP) [24] uses the AES-256 algorithm [25] to encrypt all media communications.

## 4. Implemented Systems

**Figure 3:**
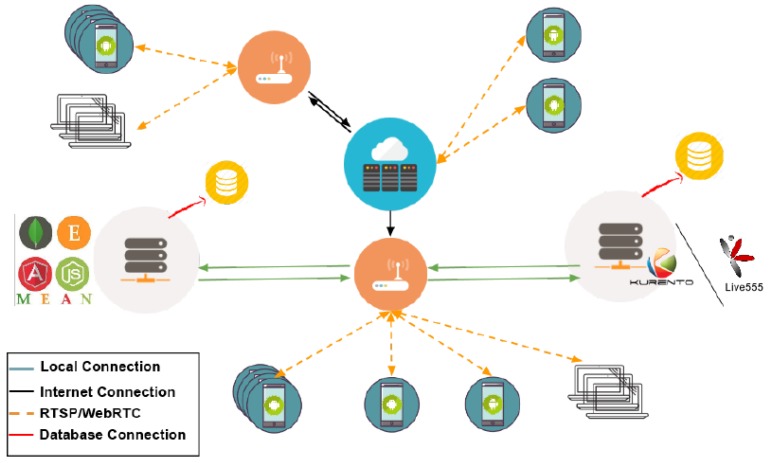
System global view.

In this work, two systems have been developed. The first one is called RTSP streaming platform because it implements the RTSP protocol. The second one is called Direct WebRTC streaming platform because it implements the WebRTC protocol. Both systems are implemented through Android applications in which the application logic and the web server application that supports the system perform different tasks. The general architecture of the two systems has been created with the same principles, changing only the protocols and the parts necessary to interact with them. The main difference between these two protocols is that while in many to many communications, when the RTSP is used, the video is only sent to the server, which resends it to the other users, in the WebRTC protocol, every user has to send the video to all other users. This happens because the WebRTC protocol is designed for peer-to-peer communications which are the scope of this work. The developed Android applications are based on the Google developer and Google design principles and using the Java language through the Android Software Development Kit (SDK), and the Native Development Kit if necessary. In conclusion, two new streaming platforms through two new Android applications have been implemented. Figure 3 shows the global architecture of the system. The systems that implement the two studied protocols have the peculiarity of using two servers each. Although these are two independent servers that interact with each other, they could be hosted on the same physical server, but for the resilience of the system, they are physically separated. These servers are the MEAN and the KURENTO or Live555 servers. The MEAN server acts as information point server that stores who is online and who is not in every moment. It also stores information about the URLs, public IPs, and other personal data about the users of the system. On the other hand, the KURENTO and Live555 servers are media servers that act as a bridge in the streaming, if necessary.

### 4.1. RTSP Streaming Platform

The architecture of the RTSP streaming platform consists of the use of P2P communications through the RTSP protocol in places where the two users are in the same network, and in the use of a Live555 media server in cases in which there are no dedicated network and Internet connection. Live555 media server is a complete open source RTSP server that supports the most common types of media files. Moreover, this server can stream multiple streams from the same or different files concurrently, using by default the UDP protocol to transmit the streams, but it is also possible to transmit over the TCP protocol if necessary.

The developed Android application acts as server and/or client depending on the application scenario. This application allows connecting with multiple users and sending their real-time stream. In the implementation of this system, it was decided to use the latest available version of Android, Android 6.0, which corresponds to the API 23 of the Android SDK. Neither this version of Android nor, consequently, in any previous, the RTSP protocol is implemented, so we had to use the third party libraries that implement this protocol. At this point, we decided to use the Libstreaming open source library that implements the RTSP protocol on both the server side and the client side. This library allows streaming the camera and/or the microphone of an Android device. It supports all devices with version equal or higher than Android 4.0, and supports H.264, H.263, Advanced Audio Coding (AAC) and Adaptive Multi-Rate (AMR) media codecs. The choice of this library is due, first of all, to the fact that this library makes a faithful implementation of the RTSP protocol. Second, this library is one of the most used libraries in the applications that implement the RTSP protocol, and, last, but not least, this library is an open source library, so that everybody can inspect it and solve any bug that may find.

Moreover, for the application to be compatible with a greater number of devices, we decided to capture the video using the MediaRecorder API that is available in the Android 4.0 version. The MediaRecorder API is not thought to be used for streaming purposes, but it can be used to get data from the peripherals of the phone. To use this method and get more potential users, we had to use a ParcelFileDescriptor to record the captured stream instead of using a file to do so. Apart from this method for recording, we could apply the MediaCodec API to record the stream using a buffer-to-buffer method or a surface-to-buffer method. To do this, it would be necessary to use the Android 4.1 or Android 4.3 versions, respectively, with the consequent loss of potential users.

**Figure 4:**
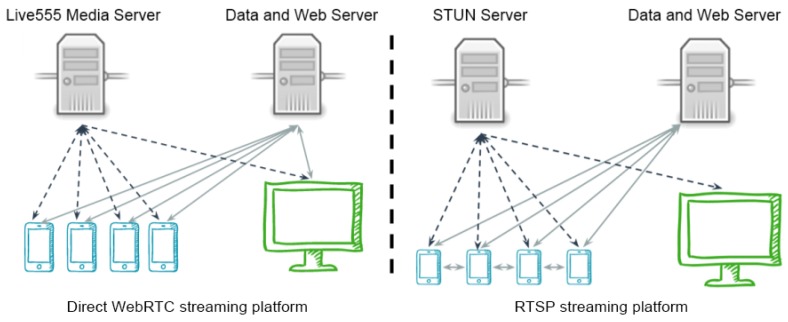
General flow of streaming platforms.

The general flow of this version of the system is now described. Firstly, when a user decides to broadcast a streaming, a request is sent to the web server starting that it will start broadcasting. The server then responds with an “OK” and the smartphone starts sending the video stream to the media server. When a user wants to see a streaming and opens the application, a request is automatically sent to the server to find out what the online streaming and its URLs are. Finally, when a user selects a streaming from the list of online streamings, the application connects to the media server, and downloads and reproduces the streaming. The first part of Figure 4 shows the general flow of the RTSP streaming platform.

### 4.2. Direct WebRTC Streaming Platform

The architecture of the Direct WebRTC platform consists of the use of P2P communications through the WebRTC protocol in places where the two users are in the same network, whereas in the cases in which the users do not have a dedicated network, the Internet connection is used.

The developed application acts as client and/or server depending on the application scenario. It allows connecting with one or multiple users and sending their real-time stream. It was decided to use the latest available version of Android, Android 6.0, which corresponds to the API 23 of the Android SDK. Neither this version of Android nor consequently in any previous, the WebRTC protocol is implemented in the core of Android, so we had to use to third party libraries that implement this protocol. At this point, it was decided to use the Libjingle library, which is an open source library written in C++ by Google to allow the establishment of P2P connections and development of P2P applications. To use this library in Android, it was necessary to compile the source code to generate the JNI API so that the Java implementation can reuse the C++ implementation of the same API. This library allows streaming the camera and/or microphone of an Android device. This library supports all devices with a version equal to or higher than Android 4.1 and supports the H.264, H.265, VP8 and VP9 media codecs. Moreover, the use of Socket.io library was also decided. This library allows us to establish communications through the Websocket protocol, so it is used in initial communications between the Android application and the Session Initiation Protocol server. The use of these libraries is because the fact that these two libraries are widely used and tested on a large number of applications and systems, both are open source libraries with a huge community supporting and testing it, and everybody can inspect and use the code and finally, the WebRTC library implements WebRTC faithfully, keeping the proposed standard. Furthermore, it was decided to use a STUN server, which is responsible for obtaining the public IP and the port used to send the video streaming.

The general flow of this version of the system is described now. First, when a user decides to broadcast, a request is sent to the server with data about the public IP and the port that this device will use to broadcast the streaming. This public IP and port are obtained through the Google STUN server. The server then answers with an “OK” response and the smartphone starts sending the video streaming to the web server. When we open the client application, a request to the server is automatically sent to find out what the online streamings and its public IP and port are. Finally, when a user selects a streaming from the list of online streaming, he/she connects to the transmitter and the streaming starts. The second part of Figure 4 shows the general flow of the Direct WebRTC streaming platform.

## 5. Comparative Study

In this section, different results obtained from the two implemented platforms are reported and used to compare their performance, both with each other, and with the most commonly used streaming applications.

In the experiments, a Lenovo G510 laptop (manufactured by Lenovo PC HK Limited, Hong Kong, China) was used as a server of the two implemented systems. This laptop has an Intel i7-4702MQ processor (manufactured by Intel Corporation, Santa Clara, CA, USA), 8 GB of RAM memory, 1 TB of storage and uses the Windows 10 × 64 operating system.

The network used to perform all the tests is made up of a TP-LINK TL-WR841N Wi-Fi router (Manufactured by TP-LINK Technologies Co., Limited, Shenzhen, China) that theoretically offers maximum transfer rate of 300 Mbps and an Internet connection of 100 Mbps, but after testing the maximum transfer rate on smartphones, the obtained maximum rate was 74.26 Mbps while the average after 10 measurements was 53.835 Mbps.

### 5.1. Comparative Analysis between the Two Implemented Systems

This subsection shows the analysis of the different measures taken on the connection establishment time and stream reception time obtained from the implementations of the two streaming protocols. The measurements were made using the system time that is implemented in Java programming language, and, therefore, in the Android platform, in the System class with the currentTimeMillis() function. This function returns the current time in milliseconds. In order to get the connection establishment time, we took the time at the moment when user A launched the streaming, and then the time again when the connection was fully established. Subtraction was then performed between them to obtain the connection establishment time. On the other hand, the stream reception time is the time between the correct communication establishment and the time when the first video packet is received.

**Figure 5:**
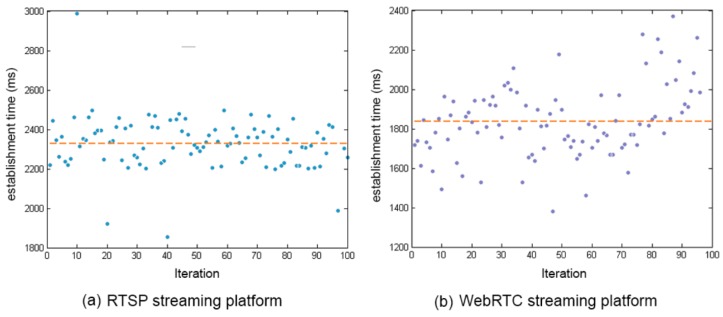
Connection establishment time.

The different measures were made using the same smartphone, an LG L9 with 1 GB of RAM memory (Manufactured by LG Electronics Inc., Yeouido-dong, Seoul, Korea), CPU 1 GHz Dual Core and 4 GB of storage memory and the 4.4.4 Android version. To perform these measurements, we implemented a series of tests using Espresso [26] tests that simulate the exact behaviour of the application. In order to obtain a significant number of measurements, the same test was performed in 100 iterations for each measurement. Figure 5 shows the comparative analysis between the two systems: RTSP and WebRTC. We can see how the system that uses the RTSP protocol is slower than the WebRTC protocol, with an average connection establishment time of 2.304 s in the case of the RTSP protocol and an average of 1.835 s in the case of the WebRTC protocol. The difference between the two implemented protocols in the average connection establishment time is 0.469. In spite of having some outliers in the plots of the two systems, WebRTC protocol is more efficient in the connection establishment time than the RTSP protocol.

**Figure 6:**
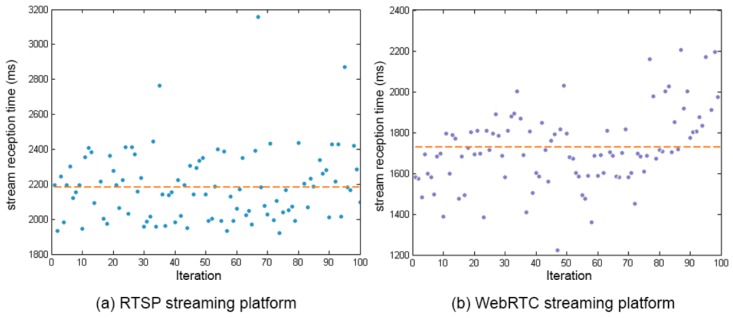
Stream reception time.

The comparison between both stream reception times can be seen in Figure 6, where a similar behaviour to the case of the connection establishment time is observed, with an average of 2.161 s for RTSP and an average of 1.709 for WebRTC. Adding the two differences, the total difference between the two protocols is almost one second to the WebRTC protocol. Moreover, we assume that the delay in the stream reception time could be repeated in other moments of the streaming, increasing the delay of the system.

**Figure 7:**
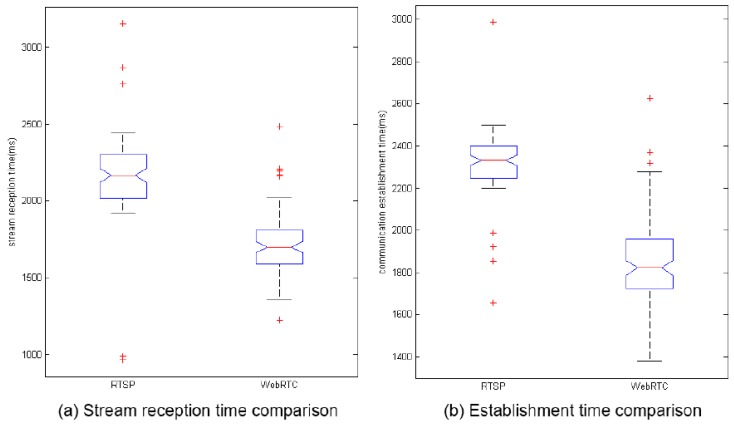
Comparison between the times of RTSP and WebRTC.

The difference between the two protocol times can be seen in Figure 7. A confidence interval of 95% has been used in all box plots. On the one hand, the first box plot in the figure shows the difference between the two protocols in the stream reception time. In this figure, a median of 2166 milliseconds, five outlier points and lower and upper limits of 1922 and 2445 milliseconds, respectively, for the RTSP streaming platform can be observed. On the other hand, the results for the Direct WebRTC streaming platform are a median of 1699 milliseconds, six outlier points and lower and upper limits of 1660 and 2026 milliseconds, respectively. The second box plot in the figure shows the difference between the two studied protocols in the communication establishment time. This figure shows a median of 2333 milliseconds, five outlier points and lower and upper limits of 2200 and 2498 milliseconds, respectively, for the RTSP streaming platform. In the other part of the plot, a median of 1822 milliseconds, three outlier points and lower and upper limits of 1380 and 2279 milliseconds, respectively, can be observed for the Direct WebRTC streaming platform. The use of these statistical values shows how the obtained measurements are grouped, which directly influences the QoS because if the measurement is not fairly grouped, the video streaming QoS could decrease, and indirectly influence the QoE in such a way that it could decrease too.

### 5.2. Comparative Analysis of Smartphone to Web Streaming Applications

On the Internet, there are many options that allow people to make video calls. In this subsection, some of the most used are analysed, and specifically the Hangouts and Facebook video streaming platforms. In order to make it possible, different measurements have been taken on the stream packet delay for Hangouts and Facebook video streaming platforms and for the two implemented systems, RTSP and Direct WebRTC streaming platforms. We used the Wireshark packet analyser in its 2.0.5 version because these applications are proprietary platforms, which means that their source code is not available and there is no protocol specification available.

To evaluate these systems, packet traces of the runs of the systems were collected and analysed. First, the control packets were separated from the data packets that interest us. Moreover, using the control data, a small overview of how these systems work can be seen. In these experiments, an amount of 10,000 data packets to be analysed were collected in order to obtain significant results. The same laptop mentioned in the previous section was used to act as server and to access the web application and the Wi-Fi network. As client, a Samsung Galaxy S6 with Exynos 7420 octa core processor (4 × 2.1 GHz Cortex-A57 & 4 × 1.5 GHz Cortex-A53) (manufactured by Samsung Electronics, Yeongtong, Suwon, Korea), 3 GB of RAM memory, 32 GB of storage memory and the 6.0 Android version was used.

The experiment consisted in making a video call between the smartphone and the web application. During this video call, communication packets were captured to be analysed using the Wireshark analyser to estimate the stream packet delay, obtained using Equation (1), where $t_{i}$ is the time when the packet *i* is received and $t_{i}-1$ is the time when the packet i−1 is received. The use of this measurement could be influenced by the codec used by the different protocols in spite of it being known that some applications use the same codecs as that the presented implementations. Moreover, the bitrate of the studied applications is not known because they are private applications, but in future works, an approximation of this aspect can be evaluated using the Wireshark packet analyser. Although these parameters can influence the measurements, the stream packet delay is a good starting point to know these applications and the possible protocols behind them because it provides an approximation to learn how the packet delay affects the different streaming platforms:

(1) $$\delta t_{i}=t_{i}-t_{i-1}.$$

Next, the stream packet delay of the two implemented platforms, RTSP and WebRTC, were estimated. The results show how the Direct WebRTC streaming platform has a much lower delay than the RTSP streaming platform. Specifically, the average delay time after 10,000 received packet is 37,807 milliseconds for the RTSP streaming platform and 8072 milliseconds for the Direct WebRTC streaming platform. These measures confirm the previous conclusion that the WebRTC implementation offers better results than the RTSP implementation.

The same analysis was performed for the Google Hangouts application. Using the captured control packets, some appointments seem pretty clear about the protocol used by this application. In the captured packets, the use of the STUN protocol can be seen, which could mean that the video streaming goes directly between the clients, without going through a media server. The use of the STUN, RTP, RTCP and SRTP protocols in this application is known because it requires having these ports open to work well. Therefore, it is likely that the video streaming protocol used in Google Hangouts is the WebRTC protocol, but we cannot be sure.

Using the data packets, the stream delay time was calculated and the obtained results are very near to those obtained by the Direct WebRTC streaming platform, which intensifies the conjecture that the WebRTC protocol is used in Google Hangout. The average measurement obtained in the stream delay time was 8.832 milliseconds.

Another analysed application was the Facebook video application. The video call feature in the Android application is out of the official Facebook application and can be found in the messenger extension application, which is the messaging application of Facebook. In the web application, the video call system can be used through the main application. In this case, like in the previous one, the use of the STUN protocol could mean that the video streaming goes directly between the clients, without going through a media server. The use of the WebRTC protocol in this application also seems clear. The obtained measurements on the media packets obtained for this application shows an average stream delay time of 11.093 milliseconds.

**Figure 8:**
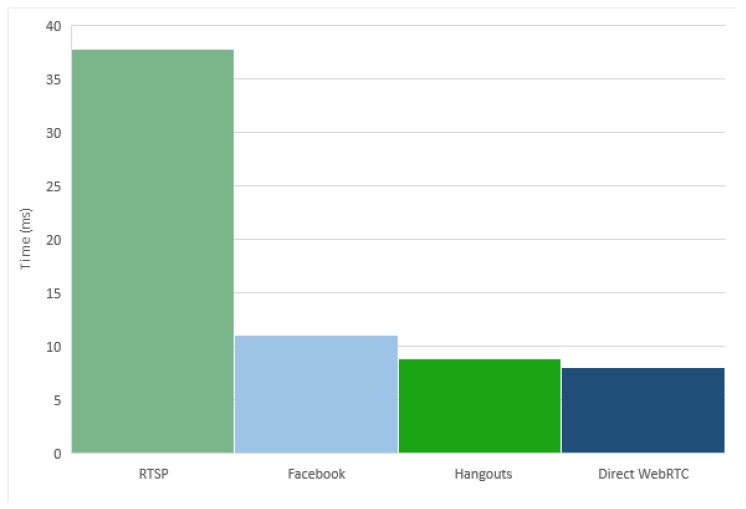
Average stream delay time.

The comparison among the average stream delays time of the four systems is shown in Figure 8, where an important improvement of the systems that implement WebRTC can be seen. In particular, the Google Hangouts and the Direct WebRTC streaming platforms have better performance than Facebook. This may be due to the use of some Google platforms as support in both systems, such as the Google STUN server.

### 5.3. Comparative Analysis of Smartphone to Smartphone Streaming Applications

The world of smartphone applications is constantly changing, and every day new applications emerge. In the case of streaming applications, it is not different. In this section, a selection of the most used and known video call applications is studied. Some applications studied in the previous section are analysed, but, in this case, the analysed communication is between two smartphones. To make this possible, different measures on the stream packet delay have been taken for Google Hangouts, Facebook, Skype and Google Duo Android applications and for the Direct WebRTC video streaming platform, the one that implements the WebRTC protocol. The Wireshark packet analyser is used because these applications are proprietary platforms, which means that their source code is not available and no specifications of the protocols are available.

In the evaluation of these systems, packet traces of the run of the systems were collected and analysed, like in the previous subsection. First, the control packets are separated from the data packets that interest us. Moreover, using the control data, a small overview on how these systems work can be seen. In these experiments, an amount of 10,000 data packets were collected and analysed to obtain significant results. The same Wi-Fi network commented in the previous section is used. As a client, a Samsung Galaxy S6 with Exynos 7420 octa core processor (manufactured by Samsung Electronics, Yeongtong, Suwon, Korea) (4 × 2.1 GHz Cortex-A57 & 4 × 1.5 GHz Cortex-A53), 3 GB of RAM memory, 32 GB of storage memory and the 6.0 Android version was used.

In the experiment, a video call was made between two smartphones using the different Android applications, collecting packets about this communication and analysing them to know the stream packet delay, which is obtained using Equation (1) of the previous subsection.

Then, the stream packet delay of the Direct WebRTC streaming platform was measured to find that it has a lower delay in the smartphone to smartphone test than in the smartphone to web application test. In particular, the average delay in this platform is 5.112 milliseconds.

For the Google Hangouts Android application, the same analysis was performed. As aforementioned, in the analysis of the captured control packets, this application seems to use the WebRTC protocol. In the captured packets, the use of the STUN protocol can be seen, and some answers of this protocol points to Google server, which could act as intermediary media server. We know that Hangouts uses the STUN, RTP, RTCP and SRTP protocols because it requires having these ports open, and if we decode the data packets, RTP data are obtained. Thus, it is probable that the used video streaming protocol is the WebRTC protocol, but we can not be sure of it.

Then, the stream delay time was calculated using the data packets. The obtained results are close to those obtained by the Direct WebRTC streaming platform, which again intensifies the conjecture that Google Hangouts use the WebRTC protocol. The obtained average stream delay time was 6.87 milliseconds.

The analysis made for the Facebook Messenger application shows that this application works in a mode similar to the previous one. After analysing all control traffic and decoding the UDP traffic as RTP, the possibility of use of the WebRTC protocol seems feasible. The data packet analysis shows a stream delay average of 16.324 milliseconds.

Another analysed application was the Skype Android application. In this case, analysing all control and data traffic, the protocol used by this application to perform the video streaming is not clear. The data communications work over the UDP protocol, but it was not possible to decode it. Thus, it is possible that Skype uses a proprietary protocol that works over UDP. The analysis of the data packets shows an average stream delay of 11.646 milliseconds.

Finally, another analysed Android application was the Google Duo video call application. In this case, the use of the WebRTC protocol is also clear. The decoding of the UDP packets as RTP packets showed different information on them, such as the sequence number, the protocol version, etc. Moreover, the use of the STUN server and the fact that the streaming video goes directly between the two smartphones, without passing through any web server, confirms the idea. In general, this implementation seems to work in a similar way to the Direct WebRTC platform. The stream delay of Duo Android application shows an average of 5.424 milliseconds.

**Figure 9:**
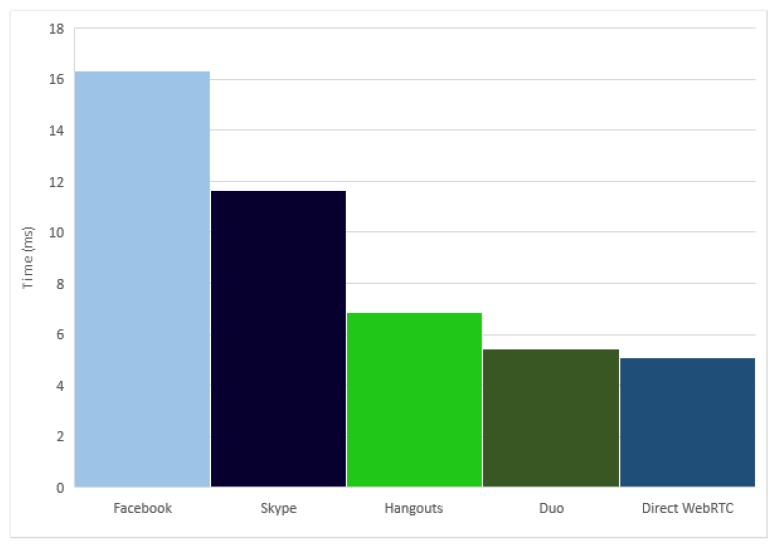
Average smartphone to smartphone stream delay time.

The average comparison among the five applications can be seen in Figure 9, where the Direct WebRTC streaming platform and the Google Duo applications show similar results due to their apparently similar implementations. They are followed closely by another Google application, the Hangouts application. Finally, Skype and Facebook applications have slower results.

### 5.4. Experiments Results Summary

The results of the experiments discussed above suggest that the use of the WebRTC protocol offers a better performance than the others, as can be seen in Table 2. In this table, we can see the average time in milliseconds of all the experiments with the different protocols and applications studied in this work. Some applications have not been used in some experiments because it is not possible to apply them using these applications, which is the reason why, in these cases, no average of them appear.

The performance improvement of the WebRTC implementation with respect to the RTSP implementation could be related to the use of UDP in all communications by the WebRTC protocol, whereas, in the RTSP protocol, TCP is used for the control. On the other hand, RTSP does not drop video packets, while the WebRTC protocol can do it if necessary. Finally, for peer-to-peer communications, WebRTC sends the video directly to the other peer, while, in the case of RTSP, the video is sent to the server and the server sends it to the other peer. Moreover, the results obtained in this study, on the improvement that the WebRTC implementation shows over RTSP in peer-to-peer communications, could be generalized because the implemented systems have faithfully implemented the standards, and use widely, tested open source libraries. Moreover, the WebRTC streaming platform shows better results than the analysed streaming applications in the stream reception time and in the stream establishment time in all cases, which means that, taking these measurements into account, the implemented streaming platform offers a better QoS than the studied applications.

## 6. Conclusions

This work includes a complete analysis of the most used video streaming protocols, paying special attention to RTSP and WebRTC protocols. Moreover, two new streaming platforms have been developed to compare both protocols and optimize their operation. These implementations have been built taking into account the most common schemes and conditions of use of Android applications. The analysis of the QoE and the QoS of both platforms was performed using two metrics: the establishment connection time and the stream reception time. From the experiments, it is concluded that significant improvements have been obtained in WebRTC over RTSP for both communication establishment time and package sending time. Moreover, the implemented systems have been compared with the most common commercial applications through two experiments. On the one hand, the new implemented platforms have been compared with the most common smartphone to web video call applications, using external software because the code of such proprietary applications is not accessible. In this experiment, both the new Direct WebRTC platform and the Hangouts application showed similar and better behaviour than the other compared systems. On the other hand, the implemented Direct WebRTC platform was compared to the most common smartphone to smartphone Android video call applications, using the aforementioned external software because the code of these proprietary applications is not accessible either. In that experiment, the implemented Direct WebRTC system again showed a good response, and together with the Google Duo application, showed the best results of the comparison. Therefore, at this point, it is possible to confirm that the use of the WebRTC protocol provides better QoE and QoS than other protocols, and that the implemented Direct WebRTC system offers good results, according to the performed experiments. This work may lead in the future to new works where the knowledge obtained in the study and implementation of the two protocols discussed here would help to study and implement adaptive streaming protocols to further improve the proposed video streaming platforms. Moreover, the study of new metrics to compare the presented streaming platforms with the commercial ones would make that the presented study could cover other aspects. Finally, a study on how video resolution and quality affect bitrate, QoE and QoS of the video streaming applications could be added when the adaptive video streaming platforms are developed.

## Figures and Tables

**Table 1:** Comparison between push-based and pull-based streaming protocols.

Feature	Push-Based	Pull-Based
Source	Broadcasters and servers like Windows media, QuickTime, RealNetworks Helix Cisco CDS/DCM	Web servers such as LAMP, RealNetworks Helix, Microsoft BS, Cisco CDS
Protocols	RTSP, RTP, UDP	HTTP (HLS, MPEG-DASH, Adobe HTTP Dynamic Streaming, Microsoft Smooth Streaming)
Bandwidth usage	Likely more efficient	Likely less efficient
Video monitoring	RTP Control Protocol (RTCP)	Currently proprietary
Multicast support	Yes	No

**Table 2:** Results of video streaming protocols and applications experiments.

	Establishment Time	Reception Time	Smartphone to Smartphone	Smartphone to Web
RTSP	2304 ms	2161 ms	37.807 ms	–
Direct WebRTC	1835 ms	1709 ms	8.072 ms	5.112 ms
Facebook	–	–	11.093 ms	16.324 ms
Hangout	–	–	8.832 ms	6.87 ms
Skype	–	–	–	11.646 ms
Duo ms	–	–	–	5.424 ms
